# TIMP-3 Alleviates White Matter Injury After Subarachnoid Hemorrhage in Mice by Promoting Oligodendrocyte Precursor Cell Maturation

**DOI:** 10.1007/s10571-024-01469-2

**Published:** 2024-04-16

**Authors:** Peiwen Guo, Xufang Ru, Jiru Zhou, Mao Chen, Yanling Li, Mingxu Duan, Yuanshu Li, Wenyan Li, Yujie Chen, Shilun Zuo, Hua Feng

**Affiliations:** 1 Department of Neurosurgery and State Key Laboratory of Trauma, Burn and Combined Injury, Southwest Hospital, Third Military Medical University (Army Medical University), 29 Gaotanyan Street, Shapingba District, Chongqing, 400038 China; 2 Department of Neurology, Xinqiao Hospital, Third Military Medical University (Army Medical University), 83 Xinqiao Main Street, Shapingba District, Chongqing, 400037 China; 3 Chongqing Key Laboratory of Precision Neuromedicine and Neuroregenaration, Southwest Hospital, Third Military Medical University (Army Medical University), Chongqing, 400038 China; 4 Chongqing Clinical Research Center for Neurosurgery, Southwest Hospital, Third Military Medical University (Army Medical University), Chongqing, 400038 China

**Keywords:** Subarachnoid hemorrhage, Pericyte, Tissue inhibitor of metalloproteinase-3, Oligodendrocyte precursor cell, White matter

## Abstract

Subarachnoid hemorrhage (SAH) is associated with high mortality and disability rates, and secondary white matter injury is an important cause of poor prognosis. However, whether brain capillary pericytes can directly affect the differentiation and maturation of oligodendrocyte precursor cells (OPCs) and subsequently affect white matter injury repair has still been revealed. This study was designed to investigate the effect of tissue inhibitor of metalloproteinase-3 (TIMP-3) for OPC differentiation and maturation. PDGFRβ^ret/ret^ and wild-type C57B6J male mice were used to construct a mouse model of SAH via endovascular perforation in this study. Mice were also treated with vehicle, TIMP-3 RNAi or TIMP-3 RNAi + TIMP-3 after SAH. The effect of TIMP-3 on the differentiation and maturation of OPCs was determined using behavioral score, ELISA, transmission electron microscopy, immunofluorescence staining and cell culture. We found that TIMP-3 was secreted mainly by pericytes and that SAH and TIMP-3 RNAi caused a significant decrease in the TIMP-3 content, reaching a nadir at 24 h, followed by gradual recovery. In vitro, the myelin basic protein content of oligodendrocytes after oxyhemoglobin treatment was increased by TIMP-3 overexpression. The data indicates TIMP-3 could promote the differentiation and maturation of OPCs and subsequently improve neurological outcomes after SAH. Therefore, TIMP-3 could be beneficial for repair after white matter injury and could be a potential therapeutic target in SAH.

**Graphical Abstract:**

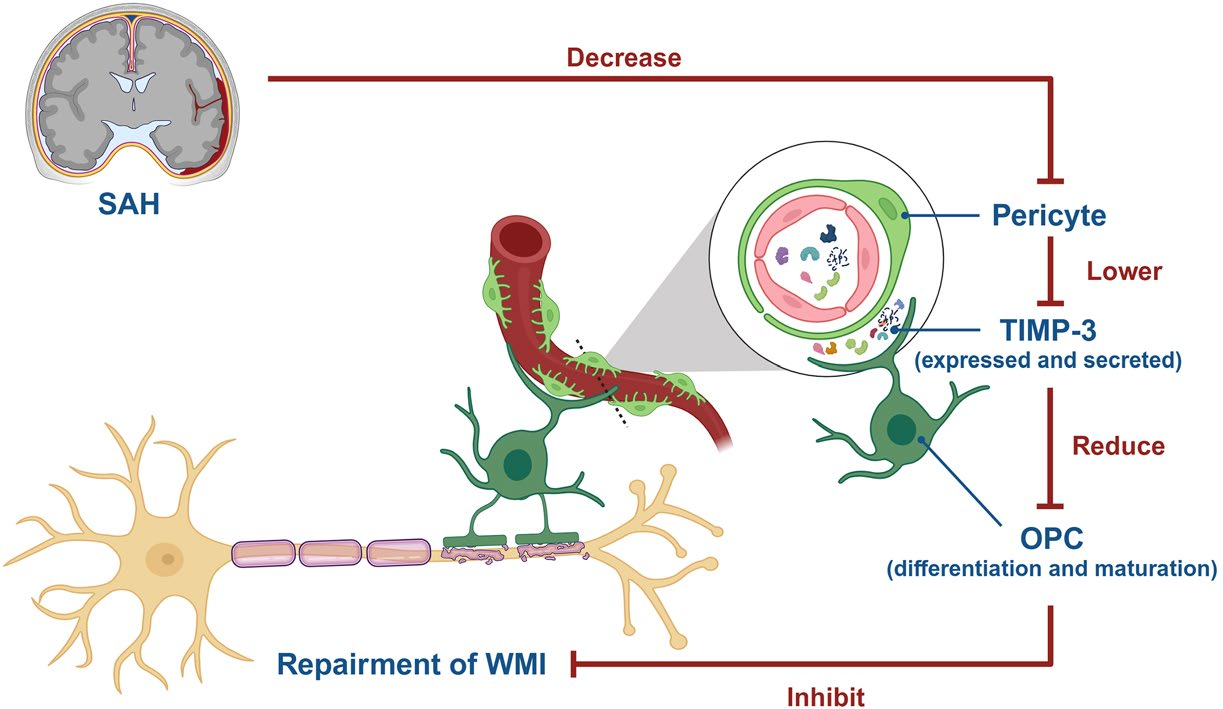

**Supplementary Information:**

The online version contains supplementary material available at 10.1007/s10571-024-01469-2.

## Introduction

Subarachnoid hemorrhage (SAH) seriously threatens human life and health due to its catastrophic consequences, such as high rates of mortality and disability and severe secondary neurological deficits. Intracranial aneurysms are the main cause of SAH. With the advancement of medical technology, intracranial aneurysms can be diagnosed and treated efficiently (D'Amato and Chang 2023). However, there is no effective treatment for secondary neurological deficits (Rost et al. 2022). A review of previous studies revealed that cerebral white matter injury plays an important role in secondary neurological deficits. The unknown mechanism of white matter injury is the main reason for the lack of prevention and treatment methods (Chen et al. 2023). Therefore, it is important to study white matter injury after SAH in depth.

Tissue inhibitor of matrix metalloproteinase-3 (TIMP-3) is a major member of the TIMP family and is mainly expressed and secreted by brain capillary pericytes. In addition, they play an important role in maintaining blood–brain barrier (BBB) integrity (Mi et al. 2021). Previous studies have shown that TIMP-3 can also regulate inflammation and extracellular matrix remodeling by specifically inhibiting MMP-3 (Mi et al. 2021; Cabral-Pacheco et al. 2020). However, whether TIMP-3 also plays an important role in the repair of cerebral white matter injury after SAH is unclear.

Oligodendrocytes (OLs) are important components of cerebral white matter. SAH can induce myelin sheath degradation and OL death through the activation of MMP-9, increased BBB permeability, decreased cerebral blood flow and direct action of blood metabolites (Peng et al. 2022). OL injury or death is the upstream and key point in the occurrence of cerebral white matter injury (Chen et al. 2014). Therefore, supplementing OLs is the main strategy for preventing white matter injury. Oligodendrocyte progenitor cells (OPCs) are located in the cerebral white matter. These materials are the main source of supplementary OLs (Li et al. 2022b). Therefore, promoting OPC maturation is key for repair after white matter injury.

In this study, we demonstrated that TIMP-3 secreted by brain capillary pericytes may play an important role in regulating the maturation of OPCs after SAH. Herein, a mouse SAH model established by endovascular perforation was used to investigate the role and mechanism of TIMP-3 in white matter injury repair.

## Methods and Materials

### Experimental Animals

All experiments involving animals were approved by the Ethics Committee of the Third Military Medical University (AMUWEC2020793). All procedures were performed in accordance with the National Institutes of Health (NIH) Guide for the Care and Use of Laboratory Animals. All the experimental mice were housed in a humidity- (50–60% relative humidity) and temperature-controlled (24 ± 1 °C) incubator with access to food and drinking water ad libitum under a 12-h light/dark cycle. Each cage usually contained five mice depending on the experimental group. The different groups of animals were not housed in the same cage. All the experimental mice received subcutaneous buprenorphine (0.02 mg/kg) within 24 h after surgery to reduce their distress and discomfort. PDGFRβ^ret/ret^ mice are an important experimental models for studying pericyte function. Compared with wild-type mice, they had significantly fewer pericytes in their brain capillaries (Moura et al. 2017). To clearly determine the relationship between TIMP-3 and pericyte, we used PDGFRβ^ret/ret^ mice as experimental subjects.

### Experimental Design

#### Experiment I

**Fig. 1:**
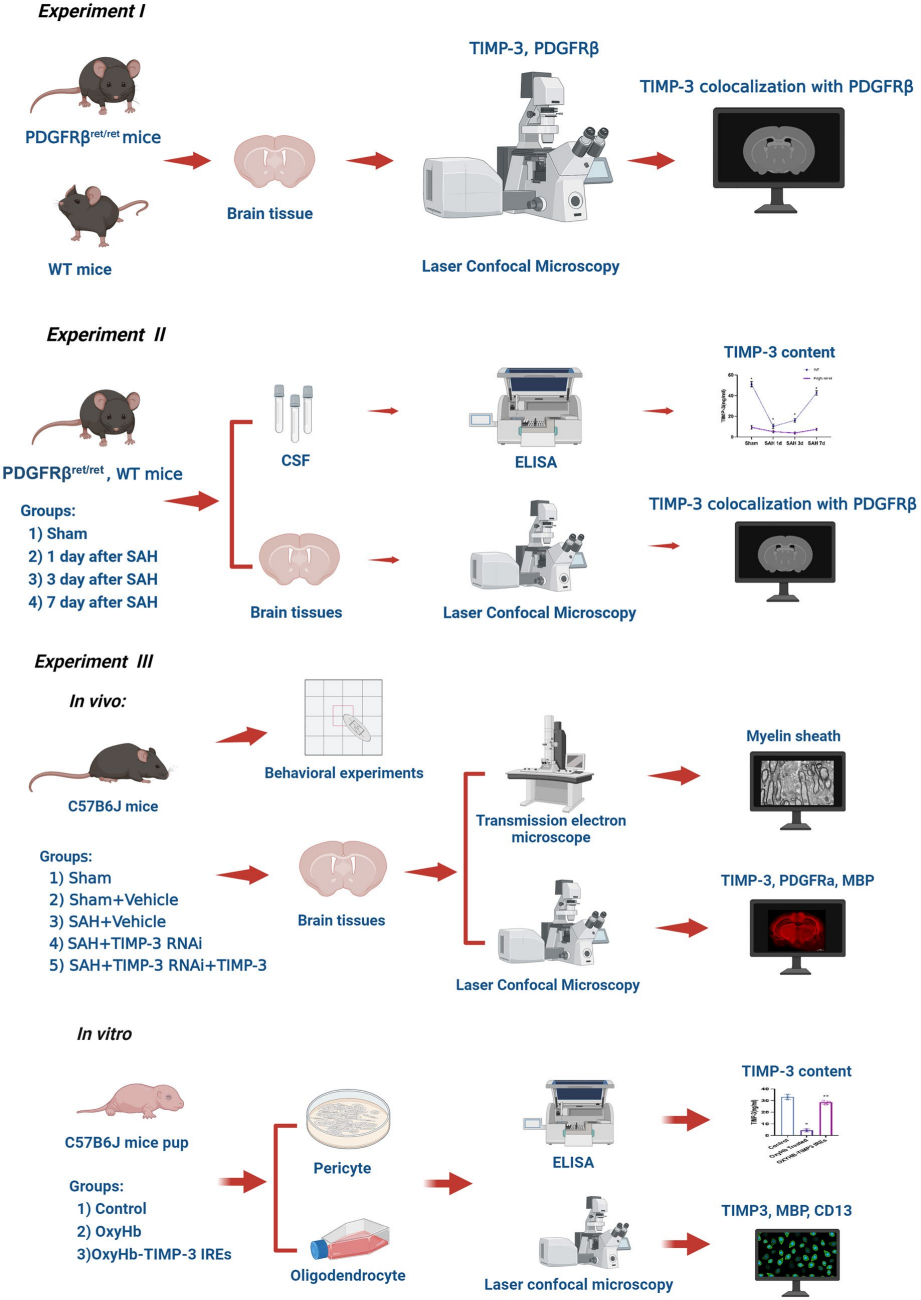
Schematic diagram of the experimental design

To investigate the relationship between TIMP-3 and pericytes, we performed immunofluorescence staining of frozen brain tissue samples from six wild-type mice and six PDGFRβ^ret/ret^ mice. The colocalization of TIMP-3 and PDGFRβ was also analyzed. The relationship between TIMP-3 and pericytes was observed and clarified by the above methods (Fig. 1*Experiment I*).

#### Experiment II

To investigate the effect of SAH on the number of pericytes and the level of TIMP-3, we randomly divided 24 PDGFRβ^ret/ret^ mice (*n* = 6) into 4 groups, namely, the sham group, the 1-day post-SAH group, the 3-day post-SAH group, and the 7-day post-SAH group. We repeated the grouping method with 24 wild-type mice. ELISA was performed to detect the content of TIMP-3 in the cerebrospinal fluid (CSF) of the experimental mice. Immunofluorescence staining and counting of TIMP-3-positive cells per mm^2^ were performed. The number of pericytes in the brain tissue of each wild-type mouse was determined. These experimental methods were applied to confirm the effect of SAH on pericytes and TIMP-3 (Fig. 1*Experiment II*).

#### Experiment III

To investigate the effect of aberrant expression of TIMP-3 on repair after SAH-induced white matter injury, we performed in vivo and in vitro experiments (Fig. 1*Experiment III*).

##### In Vivo

We randomly divided the 30 wild-type mice into 5 groups (*n* = 6): the sham group, the sham + vehicle group, SAH + vehicle group, SAH + TIMP-3 RNAi group, and SAH + TIMP-3 RNAi + TIMP-3 group. The behavioral tests included the beam balance test, determination of the modified Garcia score and the open field test. These experiments were performed at 24 h after SAH. These tests were used to assess the neurological function of the experimental mice. Changes in the myelin sheath and the content of MBP at that time were assessed by immunofluorescence staining, determination of the mean gray values, and transmission electron microscopy. Immunofluorescence staining and counting of PDGFRa-positive cells per mm^2^ were subsequently performed (PDGFRa is a marker of OPCs). The number of OPCs in the brain tissue of each experimental mouse 24 h after SAH was determined.

##### In Vitro

We used mouse primary pericytes and primary OLs as the study subjects. OxyHb and TIMP-3 overexpression and pericyte culture supernatant were used as intervention factors. ELISA, immunofluorescence staining and quantitative analysis of the number of positive cells per mm^2^ were used to detect the content of TIMP-3, as well as the number of pericytes and OLs, at 24 h after treatment.

These methods were applied to clarify the effect of TIMP-3 expression on the repair after white matter injury.

### Mouse SAH Model

We used endovascular perforation to construct a mouse SAH model(Hao et al. 2022). Briefly, all the experimental mice were anesthetized with halothane (70% N_2_O and 30% O_2_; 4% for induction, 2% for maintenance, RWD 21,081,501). A midline incision in the neck was made to expose the left external carotid artery. The vessel was ligated and severed at the distal end, leaving a 2-mm stump and blocking blood flow from the left internal carotid artery. A 5–0 sharpened monofilament nylon suture was inserted into the left internal carotid artery until slight resistance was encountered (approximately 5–12 mm). Another 1–2 mm was then inserted to penetrate the anterior and middle cerebral bifurcations. After being held in place for 10 s, the nylon suture was removed, and the left external carotid artery stump was ligated. Finally, we restored internal carotid artery blood flow (Supplemental Fig. 1a). The experimental mice in the sham group underwent the same surgical procedure as those described above, except for the punctured cerebral vessels.

### CSF Collection

We extracted CSF from the cisterna magna of the experimental mice(Siler et al. 2014). After the experimental mice were anesthetized and fixed on a stereotaxic frame, the atlas-occipital membrane was exposed through a median incision. A 10 μL Hamilton syringe (Microliter 701; Hamilton Company) was inserted (approximately 1–1.5 mm) into the cisterna magna, after which 10 μL of CSF was extracted.

### Intracerebroventricular Injection

The intracerebroventricular injection steps were performed in experimental mice as previously described (Zhou et al. 2023). Briefly, after anesthesia, the left side of the skull was drilled (coordinates: 0.6 mm posterior to the fontanelle and 1.5 mm lateral to the sagittal suture of the skull). The needle of a 10 μL Hamilton syringe was subsequently inserted 1.7 mm into the left ventricle of the experimental mice. The needle was held in place for 10 min and then slowly removed. Two types of lentiviral vectors that regulate TIMP-3 expression were used for in vivo experiments (GeneChem, Shanghai, China):

1. The Ubi-MCS-3FLAG-SV40-IREs-Puromycin lentiviral vector was used for TIMP-3 overexpression (abbreviated as the TIMP-3 IREs Group);
2. The hU6-MCS-CMV-Puromycin lentiviral vector was used for TIMP-3 interference (abbreviated as the TIMP-3 RNAi group);

All lentiviral vectors were stored at −80 °C before use. The effects of the lentiviral vectors were verified by in vitro experiments (Supplement Fig. 1b). The lentiviral vector was injected into the left lateral ventricle at 1 μL/min. One week later, the experimental mice were used to construct the SAH model. For the SAH + TIMP-3 RNAi + TIMP-3 group, mice previously injected with TIMP-3 RNAi were injected with recombinant TIMP-3 (2 µl, Novus, 973-TM-010, 1 µg/µl) through the lateral ventricle 24 h before SAH.

### Neurological Scores

At 24 h after SAH, the neurological function of the experimental mice was assessed by a double-blind method. The test included determination of the modified Garcia score and execution of the beam balance and open field tests (Qu et al. 2018; Chung et al. 2021; Zuo et al. 2017). The modified Garcia score is mainly used as a measure of spontaneous activity, symmetry of limb movement, forepaw extension, climbing, body proprioception, and response to vibrissal touch in experimental mice and ranges from 3 to 18. In the beam balance test, the experimental mice were placed in the center of a wooden beam. Then, walking distance within 1 min was assessed and scored from 0–4 points. In the open field test, the experimental mice were removed from the feeding cage and quickly placed in the central area of the experimental box. The activity of the experimental mice in the experimental box was recorded using animal behavioral analysis software for 15 min.

### Transmission Electron Microscopy

Transmission electron microscopy was used to observe the myelin sheaths of the experimental mice 24 h after SAH (Zhou et al. 2023). The experimental mice were anesthetized and sacrificed by transcardial perfusion with cold 0.9% saline. The brain tissue was removed and fixed with 4% glutaraldehyde containing 100 mmol/mL sodium cacodylate (pH = 7.3) at 4 °C overnight. Then, the brain tissue was minced into 1 mm^3^ pieces and placed in Epon (Agar 100 resin, Agar Science, Essex, UK). Afterward, the myelin sheaths of the experimental mice were observed and imaged using a transmission electron microscope (JEM-1200 EX, JEOL, Tokyo, Japan).

### Fluorescence Immunohistochemistry and Immunocytochemistry

Fluorescence immunohistochemistry was performed as previously reported (Zhou et al. 2023). In this study, experimental mice were sacrificed via transcardiac perfusion of cold saline on days 1, 3, and 7 after SAH. Then, the brain tissues were harvested, fixed, dehydrated, and sectioned using a cryomicrotome (CM3050S-3-1-1; Leica Biosystems, New York, USA). The brain tissue sections were first blocked with 0.3% Triton X-100 and 5% goat serum for 60 min at room temperature. Afterward, the brain tissue sections were incubated with diluted primary antibody overnight at 4 °C (the antibodies used were as follows: anti-PDGFRbeta antibody, Abcam #ab69506; anti-TIMP-3 antibody [20HCLC], Abcam # ab277794; mouse aminopeptidase N/CD13 antibody, R&D Systems # AF2335; and MBP-probe antibody (R29.6), Santa Cruz Biotechnology # sc-13564). The next day, the brain tissue sections were incubated with the corresponding fluorescent secondary antibody for 2 h at room temperature. Later, the nuclei were stained with DAPI for 5 min. The expression and distribution of different protein markers were observed via laser confocal microscopy (LSM880, Carl Zeiss, Oberkochen, Germany). Representative brain tissue sections from each experimental group were obtained for imaging. Immunofluorescence images were analyzed using ImageJ V1.53 (NIH) to determine the fluorescence intensity and number of positive cells.

Immunocytochemistry was also performed as previously reported (Zhou et al. 2023). The cultured experimental cells were first fixed with 4% paraformaldehyde for 15 min. Afterward, the impurities and paraformaldehyde around the cells were removed by washing with PBS. Primary antibodies (anti-TIMP-3 antibody [20HCLC], Abcam # ab277794; mouse aminopeptidase N/CD13 antibody, R&D Systems # AF2335; MBP-probe antibody [R29.6], Santa Cruz Biotechnology # sc-13564); secondary antibodies; and DAPI staining were used. The following steps were performed for fluorescence immunohistochemistry as described above.

### Cell Culture and Treatments

Primary pericytes were cultured following previously reported procedures (Zhou et al. 2023). In brief, the steps were as follows: 1. Brain tissue was obtained from C57B6J mice (1 to 3 days old). 2. The cells were digested with MEM, HEPES (A501612, Sangon Biotech, Shanghai, China) and 40 μg/mL DNase I for 70 min. An additional 30% bovine serum albumin (A8010, Solarbio, Beijing, China) was added to the PBS for the final digestion step (BSA:PBS = 1.7:1). 3. The samples were then centrifuged at 1360 × g for 10 min. The pellet was carefully collected and incubated in EGM-2MV BulletKit medium (CC-3202, Lonza Bioscience, Basel, Switzerland). The cells were subsequently resuspended. The samples were subsequently centrifuged at 250 × g for 5 min. 4. Primary pericytes were subsequently seeded in culture dishes (C8061; Solarbio, Beijing, China) at a density of 5 × 10^5^/cm^2^ and cultured in an incubator. 5. The culture medium was changed after 20 h and then every 3 days thereafter. When the primary pericytes formed a confluent layer, three consecutive cell passages were performed at a 1:4 ratio. 6. After the third generation, the primary pericytes were cultured in pericyte medium (e76127787; ScienCell Research Laboratories, California, USA). Primary pericytes were subsequently subjected to in vitro experiments.

Primary OLs were cultured following previously reported procedures (Li et al. 2022a). In brief, the steps were as follows: 1. Brain tissue was obtained from C57B6J mice (1 to 3 days old). 2. The cerebral cortex was separated and cut into 2–3 mm^2^ pieces. 3. Then, brain tissue digestion was performed with papain and DNase (Sigma‒Aldrich, Darmstadt, Germany) in a 37 °C water bath for 10 min with occasional inversion. 4. The obtained cells were centrifuged at 1000 × g for 5 min and resuspended in growth medium supplemented with 90% DMEM/F12 (Gibco, Massachusetts, USA), 10% fetal bovine serum (Gibco, Massachusetts, USA) and 1% penicillin streptomycin (Solarbio, Beijing, China). 5. The isolated cells were maintained in T-75 culture flasks (Solarbio, Beijing, China). 6. The culture medium was changed once every 3 days. On day 6, the culture medium was changed to OL differentiation medium. 7. The OL differentiation medium was changed every 3 days. On day 9, OLs were obtained and purified for use in in vitro experiments.

For in vitro experiments, primary pericytes were incubated with OxyHb (Sangon Biotech, Shanghai, China) for 24 h to mimic SAH. The Ubi-MCS-3FLAG-SV40-puromycin lentiviral vector was used to upregulate TIMP-3 expression in pericytes (TIMP-3 RNAi group). The pericytes were infected (MOI = 10) for 24 h, transferred back to conventional medium and cultured for 72 h before the experiments were performed.

#### ImageJ Was Used for Image Analysis

Immunofluorescence images of each group taken under the same magnification and exposure conditions were collected. The immunofluorescence images taken were imported into ImageJ software. To reduce the impact of background noise, background subtraction was applied to each image. By selecting the average intensity of the undyed area in the image, this intensity value was subtracted from the entire image. ImageJ’s threshold tool was used to select fluorescently labeled areas in the image. We subsequently adjusted the threshold to include all positive fluorescence signals while excluding nonspecific signals. For each image, we manually mapped the area of interest, ensuring that only specific parts of the cell or tissue were analyzed for fluorescence intensity. Afterward, using ImageJ’s “Measure” function, the average fluorescence intensity within each ROI was calculated, as was the corresponding region size. The fluorescence intensity data and region sizes of all ROIs were exported into spreadsheet software for further analysis. All the imaging data in each experimental group were statistically analyzed. The mean fluorescence intensity and standard deviation were determined. To ensure the accuracy of the analysis, all the quantitative steps were performed independently by two researchers. The results were compared and validated. In our Results section, we reported the mean fluorescence intensities and their standard deviations for all the experimental groups. Afterward, we compared the results using appropriate statistical methods.

### Statistical Analysis

All the data were statistically analyzed and plotted using Prism 9 software. The required sample size for each experimental group is determined by ‘http://powerandsamplesize.com/’. Calculated using online tools and used as the basis for research design, Shapiro‒Wilk tests were performed on the data from each experimental group. P values greater than 0.05 were considered to not significantly violate the normality hypothesis. To determine the homogeneity of the variance between the experimental groups, we performed an F test. A P value greater than 0.05 indicated that there was no variance heterogeneity. These tests were all conducted using Prism 9 (GraphPad). The results of the tests determined the statistical methods used for subsequent data analysis. All the data are presented as the means ± standard deviations and were analyzed using Prism 9 (GraphPad) software. Groups were compared using one-way ANOVA + Tukey’s multiple comparison test. Neurological scores were analyzed by using the χ2 test. Two-way repeated-measures ANOVA was used to compare the behavioral data of TIMP-3 levels between different time points and different groups. Differences were considered statistically significant at *P* < 0.05.

## Results

### Animal Mortality

A total of 122 adult male mice (8–12 weeks, 25–30 g, provided by the Laboratory Animal Center, Army Military Medical University, Chongqing, China) were used in this study. Of these, 17 experimental mice with mild SAH scores (7 of which were PDGFRβ^ret/ret^ mice) and 13 experimental mice (three of which were PDGFRβ^ret/ret^ mice) were excluded from the study due to death within 24 h after SAH. Twelve C57B6J mice (1 to 3 days after SAH) were used for in vitro experiments. No significant differences in mortality rate were observed between the experimental groups.

### TIMP-3 is Mainly Expressed and Secreted by Pericytes

**Fig. 2:**
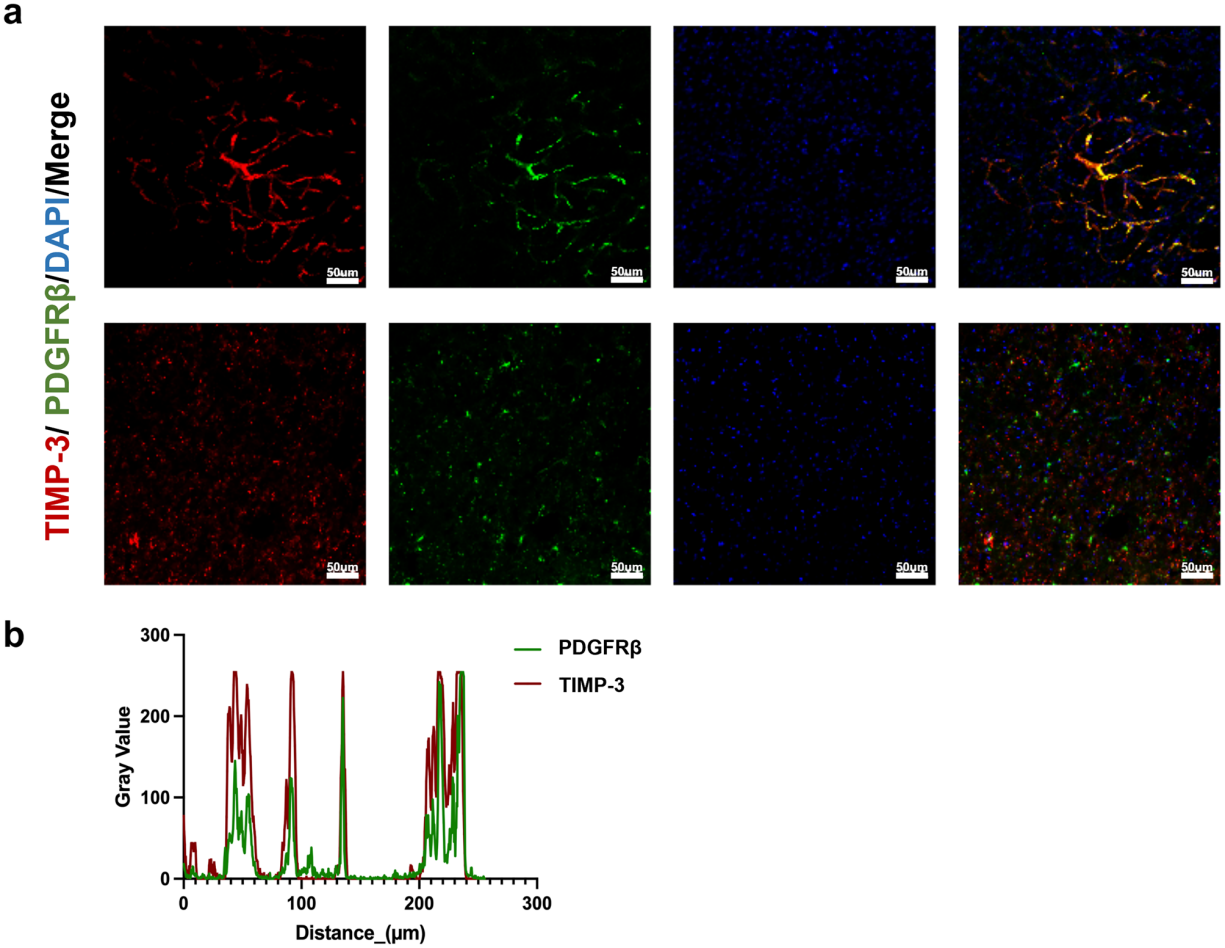
Expression and localization of TIMP-3 in pericytes. (**a**) Immunofluorescence staining for TIMP-3 (red) and PDGFRb (green) and DAPI (blue) in the white matter of the PDGFRβ^ret/ret^ mice and WT mice (*n* = 6 per group; scale bar = 50 µm). (**b**) Colocalization analysis of TIMP-3 and PDGFRβ expression showed that TIMP-3 was mainly expressed and secreted by pericytes

To verify the relationship between TIMP-3 and pericytes, we performed immunohistochemical staining of brain sections from PDGFRβ^ret/ret^ mice (Abramsson et al. 2003) and wild-type mice. By comparison, we found that, compared with those in wild-type mice, the pericyte density in the brain capillaries of the PDGFRβ^ret/ret^ mice was significantly lower. These findings were consistent with the expression and secretion of TIMP-3 (Fig. 2a). Colocalization analysis of TIMP-3 and PDGFRβ expression also confirmed that TIMP-3 was mainly expressed and secreted by brain capillary pericytes (Fig. 2b).

### TIMP-3 Content Decreased Significantly After SAH

**Fig. 3:**
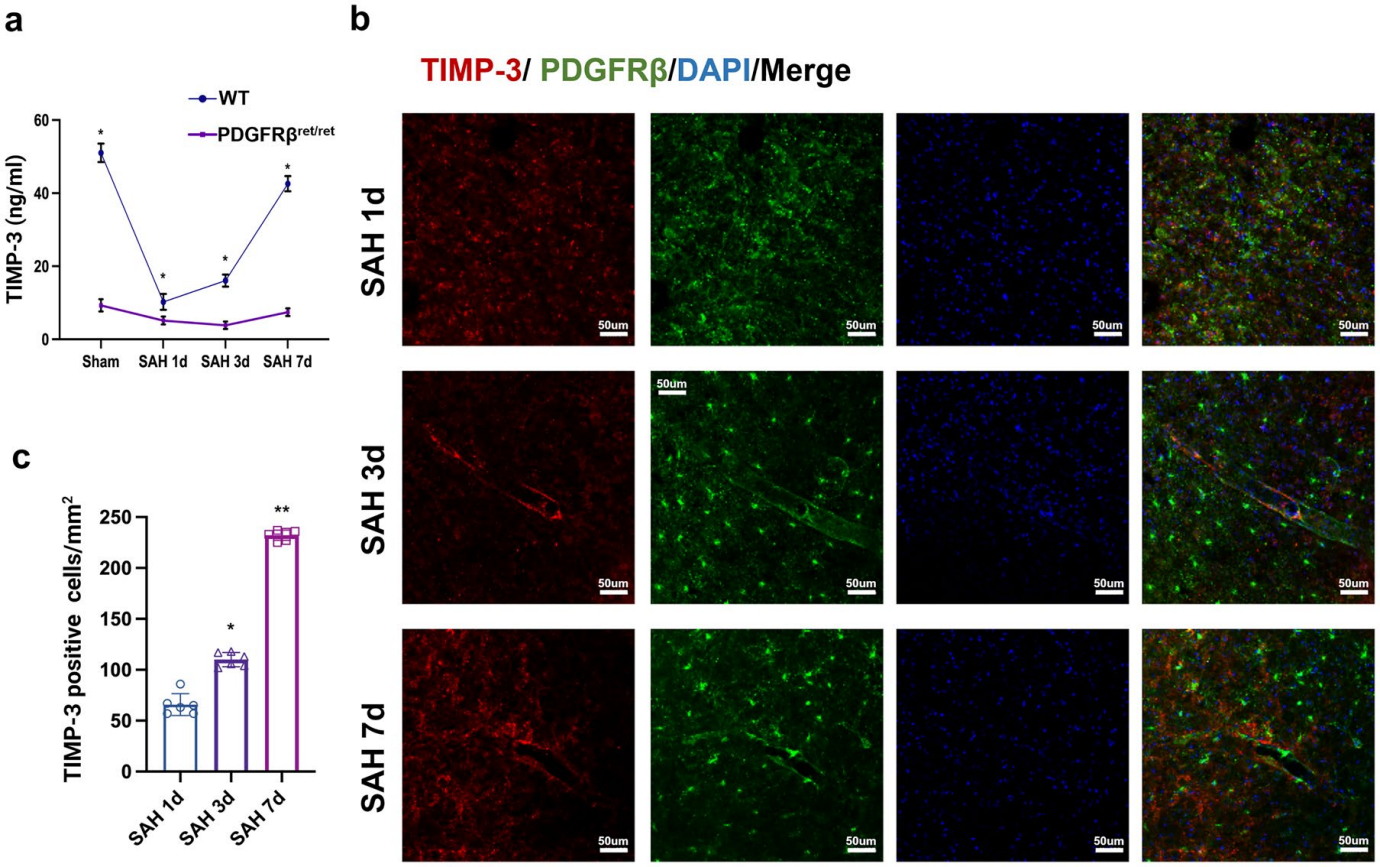
TIMP-3 content significantly decreased after SAH. (**a**) TIMP-3 levels in experimental mouse CSF were measured via ELISA (*n* = 6 per group). (* vs. PDGFRβ^ret/ret^ group, two-way ANOVA, row factor: F (3, 6) = 254.9, *P* < 0.0001; column factor: F (1, 2) = 7557, *P* = 0.0001). (**b**) Immunofluorescence staining for TIMP-3 (red) and PDGFRb (green) and DAPI (blue) in the white matter of WT mice in the 1-day post-SAH, 3-day post-SAH, and 7-day post-SAH groups (*n* = 6 per group; scale bar = 50 µm). (**c**) Number of TIMP-3-positive cells per mm^2^ in WT mice after SAH (*n* = 5 per group). (* vs. 1-day post-SAH group; ** vs. 3-day post-SAH group; one-way ANOVA; F (2, 15) = 714.1, *P* < 0.0001)

To elucidate the effect of SAH on the TIMP-3 protein concentration, we extracted the CSF of the experimental mice for ELISA. We found no significant changes in the TIMP-3 concentration in the CSF of the PDGFRβ^ret/ret^ mice after SAH (Fig. 3a). However, the content of TIMP-3 in the CSF of the WT mice was significantly greater than that in the CSF of the PDGFRβ^ret/ret^ mice in the sham group, 1-day post-SAH group, 3-day post-SAH group, and 7-day post-SAH group (Fig. 3a). Among the WT mice, the TIMP-3 content was the lowest at 1 day after SAH and then increased slowly on days 3 and 7. Immunohistochemical staining of the WT mouse brain tissue revealed that the number of TIMP-3-positive cells in mm^2^ was the lowest on day 1 after SAH (Fig. 3a, 3b). Subsequently, it increased slowly (Fig. 3a, b). Moreover, the number of TIMP-3-positive cells on day 3 after SAH significantly differed from that on day 1 after SAH (Fig. 3c). There was a significant difference in the number of TIMP-3-positive cells on day 7 versus day 3 after SAH (Fig. 3c).

### TIMP-3 Could Affect the Neurological Function of Experimental SAH Mice

**Fig. 4:**
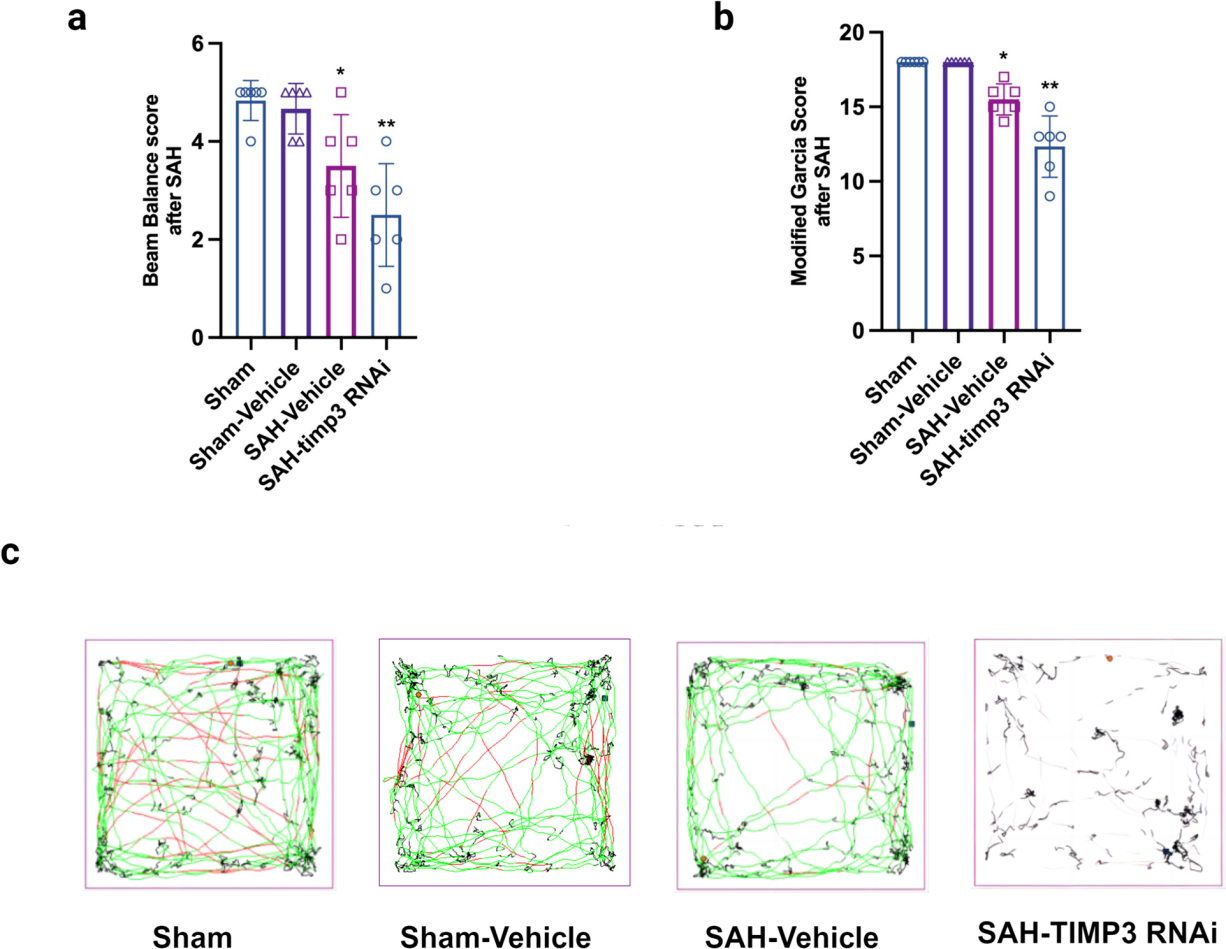
Effect of TIMP-3 concentration on neurological scores. (**a**) The modified Garcia scores of each group on day 1 after SAH (*n* = 6 per group). (* vs. sham group; ** vs. SAH-TIMP-3 RNAi group; one-way ANOVA; F (2, 15) = 27.05, *P* < 0.0001). (**b**) Beam balance test results for each group on day 1 after SAH (*n* = 6 per group). (* vs. sham group; ** vs. SAH-TIMP-3 RNAi group; one-way ANOVA; F (2, 15) = 10.42, *P* = 0.0015). (**c**) Open field test results for each group on day 1 after SAH (n = 6 per group)

To explore the effect of TIMP-3 on neurological function in experimental mice, we evaluated the modified Garcia score and performed beam balance and open field tests. Compared with those in the sham group, the modified Garcia score (Fig. 4a), beam balance (Fig. 4b), and open field test (Fig. 4c) results were significantly different between the SAH + vehicle group and the SAH + TIMP-3 RNAi group. Meanwhile, the differences in the sham + vehicle group, are not so obvious compared with those in the sham group. Moreover, the decrease in the SAH + TIMP-3 RNAi group was more significant than that in the SAH + vehicle group (Fig. 4a, b, c).

### TIMP-3 Affected the White Matter of the Experimental SAH Mice

**Fig. 5:**
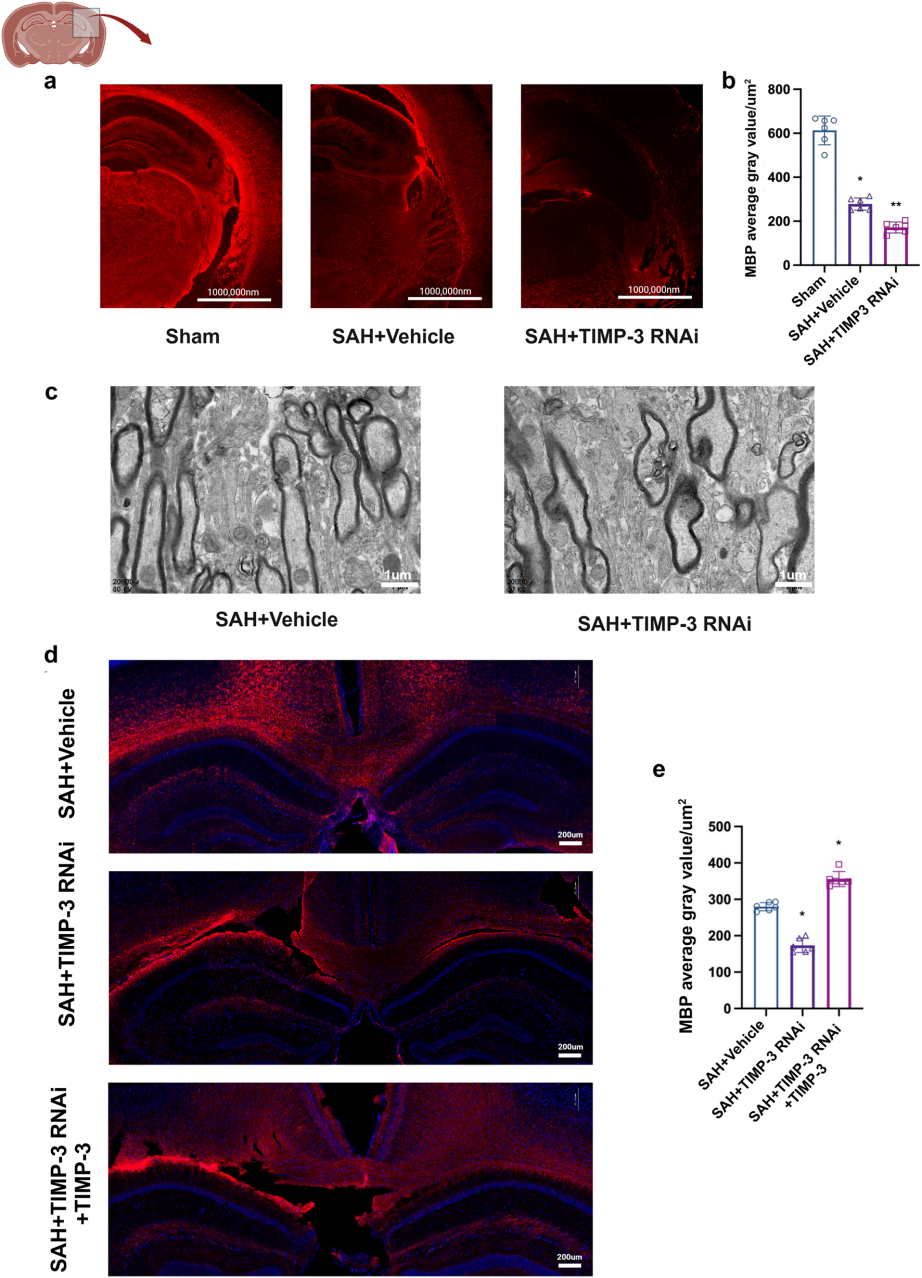
Effect of TIMP-3 content on white matter. Immunofluorescence labeling of MBP (red) in the brain tissue of each experimental group on day 1 after SAH (*n* = 6 per group; scale bar = 1,000,000 nm). (**b**) Average gray value of MBP per μm2 in each experimental group on day 1 after SAH (*n* = 6 per group). (* vs. sham group; ** vs. SAH-vehicle group; one-way ANOVA; F (2, 15) = 169.0, *P* < 0.0001). (**c**) Transmission electron microscopy was used to observe myelin sheath damage in each group on day 1 after SAH (*n* = 6 per group; scale bar = 1 µm). (**d**) Immunofluorescence labeling of MBP (red) in the brain tissue of each experimental group on day 1 after SAH (*n* = 6 per group; scale bar = 200 µm). (**e**) Average gray value of MBP per μm^2^ in each experimental group on day 1 after SAH (*n* = 6 per group). (* vs. SAH + Vehicle group, one-way ANOVA, F (2, 15) = 188.5, *P* < 0.0001)

Neurological deficits are closely related to white matter injury after SAH. We used immunohistochemical staining, the mean gray values of MBP per unit area, and transmission electron microscopy to assess the extent of white matter injury. Compared with that in the sham group, the MBP content in both the SAH + vehicle and SAH + TIMP-3 RNAi groups was significantly decreased (Fig. 5a). Moreover, compared with that in the SAH + vehicle group, the MBP content in the SAH + TIMP-3 RNAi group was significantly decreased (Fig. 5b). Transmission electron microscopy revealed that myelin sheath damage was more severe in the SAH + TIMP-3 RNAi group than in the SAH + vehicle group (Fig. 5c). However, the SAH + TIMP-3 RNAi + TIMP-3 group exhibited a significant increase in the content of MBP (Fig. 5d, e).

### TIMP-3 Affects OPCs in Experimental Mouse Brain Tissue

**Fig. 6:**
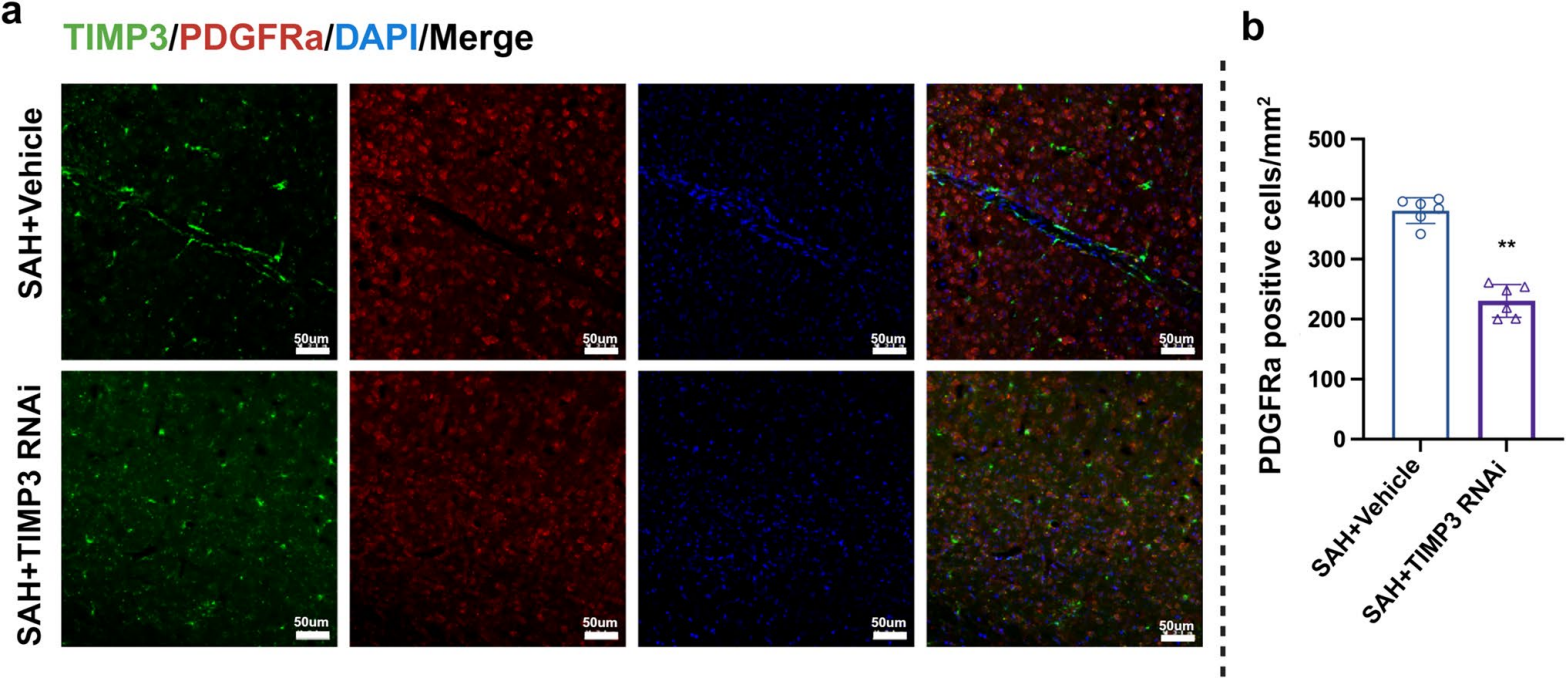
Effect of TIMP-3 content on OPCs in experimental mice after SAH. (**a**) Immunofluorescence staining for TIMP-3 (green) and PDGFRa (red) and DAPI (blue) in the white matter of each experimental group on day 1 after SAH (*n* = 6 per group; scale bar = 50 µm). (**b**) Number of PDGFRa-positive cells per mm^2^ in experimental mice at 1 day after SAH (*n* = 6 per group). (** vs. SAH-vehicle group, *t* test, *t*(10) = 10.58, *P* < 0.0001)

To elucidate the effect of TIMP-3 on OPCs after SAH, we performed immunohistochemical staining on brain tissue sections (Fig. 6a). We also counted PDGFRa-positive cells per unit area (PDGFRa is a marker of OPCs). Compared with that in the SAH + vehicle group, the number of PDGFRa-positive cells was significantly lower in the SAH + TIMP-3 RNAi group on day 1 after SAH (Fig. 6b).

### Upregulation of TIMP-3 Expression Reduced Pericyte Damage In Vitro

**Fig. 7:**
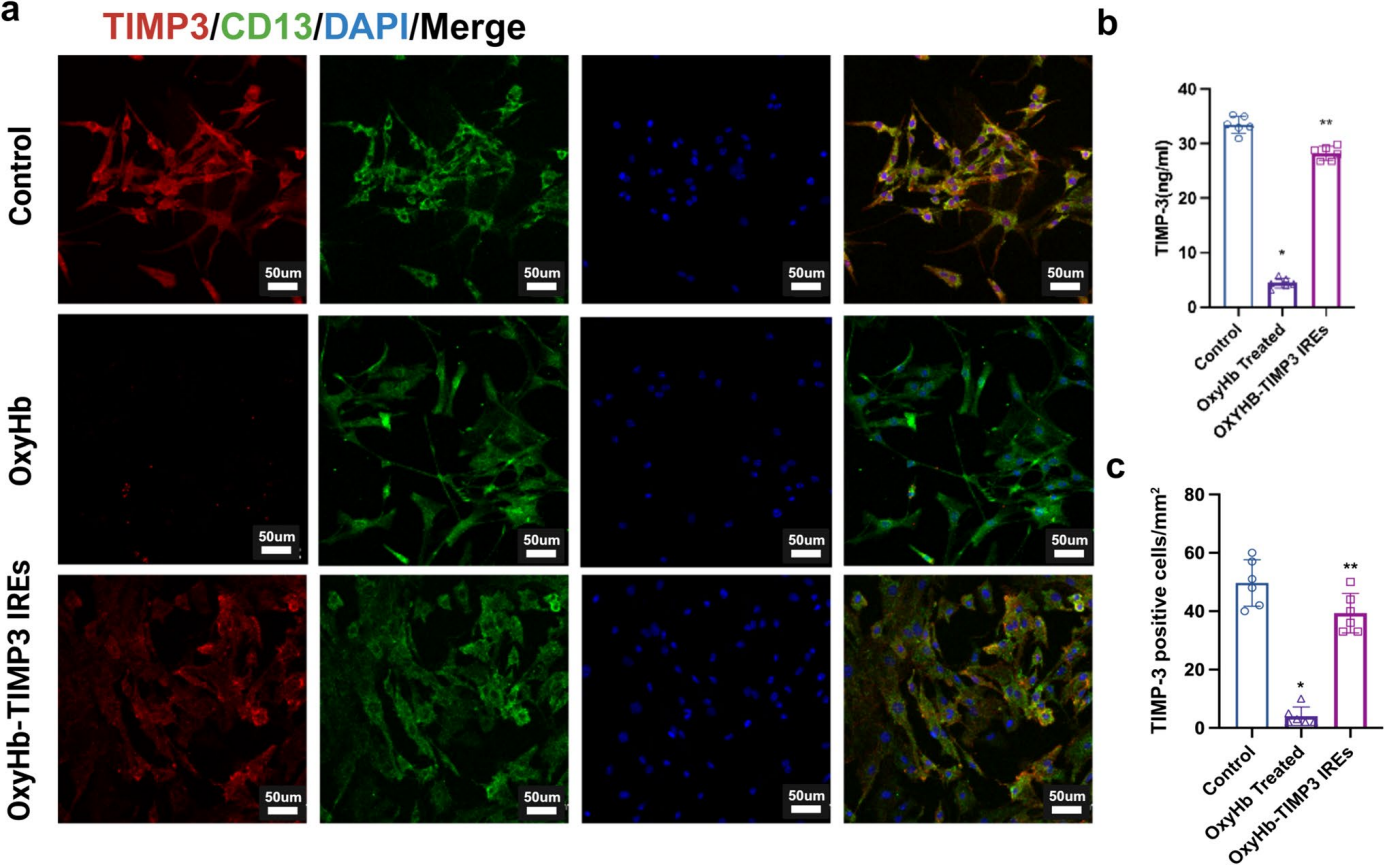
Effect of TIMP-3 content on primary pericyte damage induced by OxyHb. (**a**) Immunofluorescence staining for TIMP-3 (red) and CD13 (green) and DAPI (blue) in primary pericytes from each experimental group (*n *= 6 per group; scale bar = 50 µm). (**b**) TIMP-3 levels in the cell culture supernatant of each group were measured via ELISA (*n* = 6 per group). (* vs. control group; ** vs. OxyHb-treated group; one-way ANOVA; F (2, 15) = 93.32, *P* < 0.0001). (**c**) Number of TIMP-3-positive cells per mm^2^ in each experimental group (*n* = 6 per group). (* vs. control group; ** vs. OxyHb-treated group; one-way ANOVA; F (2, 6) = 237.6, *P* < 0.0001)

To elucidate the role of TIMP-3 in SAH, we targeted primary pericytes and used OxyHb as an intervention factor. We found that OxyHb significantly downregulated TIMP-3 expression (Fig. 7a, b) and affected the number of pericytes compared with those in the control group (Fig. 7c). However, the *TIMP-3* gene was overexpressed in the OxyHb-TIMP-3 IRE group, which increased the content of TIMP-3 in the cell culture supernatant (Fig. 7b) and the number of TIMP-3-positive cells per unit area (Fig. 7c). This resulted in a significant difference compared with that in the OxyHb group.

### Pericytes Upregulated the Expression of the MBP Protein in OLs

**Fig. 8:**
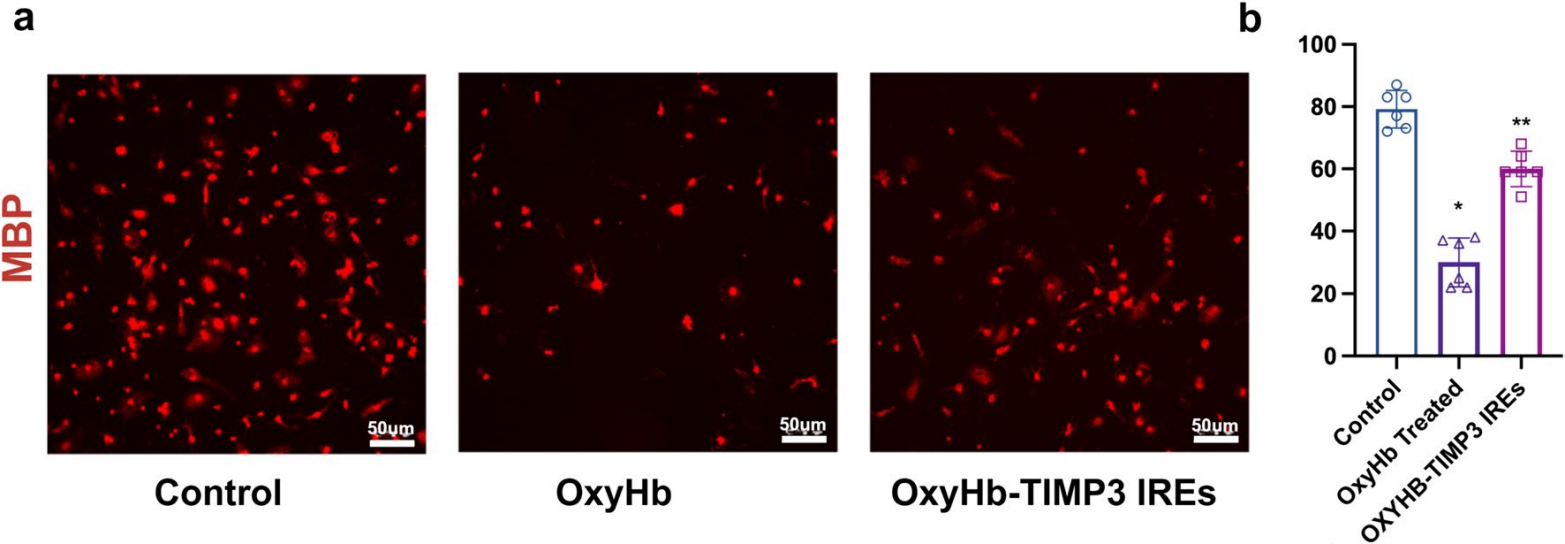
Effect of TIMP-3 content on primary OL damage induced by OxyHb. Immunofluorescence labeling of MBP (red) in the primary OLs of each experimental group (*n* = 6 per group; scale bar = 50 µm). (**b**) Number of MBP-positive cells per mm^2^ in each experimental group (*n* = 6 per group). (* vs. control group; ** vs. OxyHb-treated group; one-way ANOVA, F (2, 15) = 84.92, *P* < 0.0001)

To elucidate the relationship between pericytes and OLs, we treated mouse primary OLs with supernatants from different experimental groups. Compared with that in the control group, the protein expression of MBP in the OLs in the OxyHb group was significantly lower (Fig. 8a). However, the supernatant from the OxyHb-TIMP-3 IRE group significantly upregulated the protein expression of MBP in OLs (Fig. 8b).

## Discussion

This study aimed to evaluate the role of TIMP-3 in SAH-induced white matter injury repair. Related experiments revealed that TIMP-3 was expressed and secreted mainly by brain capillary pericytes. SAH significantly downregulate the number of pericytes and reduced the content of TIMP-3. By combining in vivo and in vitro experiments, we found that TIMP-3 plays an important role in the differentiation and maturation of OPCs after SAH. However, interfering with the expression and secretion of TIMP-3 via RNAi and lentiviral vectors can regulate the number of OPCs and the content of MBP after SAH. Therefore, it can affect repair after white matter injury and improve secondary neurological deficits.

The white matter is mainly composed of OLs, myelin sheaths formed by OL processes, and neuronal axons wrapped by myelin sheaths. Many studies have shown that white matter injury can lead to different degrees of attention impairment, memory decline, slow thinking and emotional changes (Lauzier et al. 2023). SAH can induce myelin sheath degradation and OL death through MMP-9 activation, increased BBB permeability, reduced cerebral blood flow and direct blood metabolite action. Thus, it plays a crucial role in secondary neurological deficits (Chen et al. 2022). Therefore, supplementing missing OLs has become the main strategy for preventing white matter injury. OPCs located in white matter are the main source of supplementary OLs. Normally, OPCs are relatively stationary, but OPCs can be rapidly activated when acute damage to the central nervous system occurs (Li et al. 2018). OPC activation is mainly manifested by an increased cell number, morphological changes (such as cell enlargement and synaptic hyperplasia), and increased expression of marker proteins (Li et al. 2018, 2022b). Our previous study showed that insufficient OPC activation was a vital factor for the persistence of white matter injury (Chen et al. 2014), but the cause of this underactivation was not determined. Therefore, in the present study, we experimentally confirmed that SAH-induced damage to pericytes might be an important cause of insufficient OPC activation.

In the central nervous system, pericytes have a high-distribution density. In addition to their important role in regulating microcirculation, they can also make direct contact with a variety of cells near the BBB (Solar et al. 2022). Significant changes in cerebral capillary pericyte number, morphology, and function can occur after SAH. Overall, SAH can reduce the number of pericytes; in terms of morphology, pericytes change from flat to spherical. In terms of function, SAH mainly affects contractility, immunity and phagocytes, vasculogenesis, and BBB function (Yan et al. 2021; Zhou et al. 2023). Previous studies have shown that after SAH, hemoglobin induces changes in the pericyte phenotype by inhibiting the NO/cGMP signaling pathway, resulting in sustained and strong contraction of brain capillaries (Li et al. 2016). This abnormal contraction can affect the blood flow to the cerebral microcirculation. The cerebral microcirculation can subsequently cause white matter injury (Li et al. 2016; Yan et al. 2021). However, whether pericytes play a direct role in white matter injury after SAH is unclear. Pericytes are not in direct contact with either OPCs or OLs. In this study, we found that TIMP-3 secreted by pericytes had a direct effect on both OPCs and OLs. This finding coincides with the idea that pericytes can regulate neighboring cells via their own secretory functions (De La Fuente et al. 2017).

The role of TIMP-3 in SAH has not been studied intensively and is thus not fully understood. Previous studies have suggested that TIMP-3 might play a role in maintaining BBB integrity and regulating brain capillary tube diameter after SAH. In blood samples from SAH patients, TIMP-3 levels gradually decreased to a minimum during the early period (24–72 h after SAH) and gradually increased during the delayed period (4–15 days after SAH) (Fischer et al. 2013). In this study, we tested the CSF of experimental mice. We subsequently found that the changes in TIMP-3 content were similar to the above results and that the content of TIMP-3 was positively correlated with neurological function scores after SAH. Further studies confirmed that the content of TIMP-3 after SAH was closely correlated with the number of pericytes, OPCs and OLs and affected repair after white matter injury. However, previous studies have suggested that mice have a faster metabolic rate than humans. Why did TIMP-3 decline in the CSF of experimental mice at the same rate as in humans? This difference might be related to the effect of SAH on CSF circulation. These results update the understanding of the function of TIMP-3 and lay a solid foundation for further research.

While this study adds to our understanding of TIMP-3 function and the mechanism of white matter injury after SAH, it also has several limitations. First, the specific mechanism by which TIMP-3 regulates OPC activation has not been elucidated due to changes in the field. Nevertheless, considering our findings and those of previous studies, we believe that the PAR-1 receptor in OPCs may be the target of TIMP-3. Second, we failed to simulate the effect of pericytes on OPCs in vitro due to unsolved problems in OPC culture. Thus, the effect of pericytes on OPCs was confirmed only by in vivo experiments. Finally, we also did not clarify how TIMP-3 allows activated OPCs to mature into OLs. These deficiencies will be clarified and confirmed in future studies.

## Conclusion

In the present study, SAH downregulated the expression of TIMP-3 in pericytes, which subsequently affected OPC activation and maturation into OLs, leading to repair failure after white matter injury and secondary neurological deficits. However, the upregulation of TIMP-3 expression reversed these effects. Therefore, TIMP-3 may be a new key target for the treatment of white matter injury after SAH.

## Supplementary Information

Below is the link to the electronic supplementary material.

Supplemental Fig. 1 SAH score diagrammatic sketch and TIMP-3 mRNA levels. (**a**) SAH score diagrammatic sketch. (**b**) The expression level of TIMP-3 mRNA was detected by PCR (*n*=6 per group). (* vs. control group; one-way ANOVA, F (3, 24) = 4189, *P*<0.0001) (TIF 897 KB)

## Data Availability

All data generated or analyzed during this study are included in this published article and supplemental materials. The datasets used and/or analyzed during the current study are available from the corresponding author on reasonable request.
