# Supplementary figures and images for: TIMP-3 Alleviates White Matter Injury After Subarachnoid Hemorrhage in Mice by Promoting Oligodendrocyte Precursor Cell Maturation
Source: Cell Mol Neurobiol. 2024 Apr 16;44:33. doi: 10.1007/s10571-024-01469-2 (PMC11021342; doi:10.1007/s10571-024-01469-2)

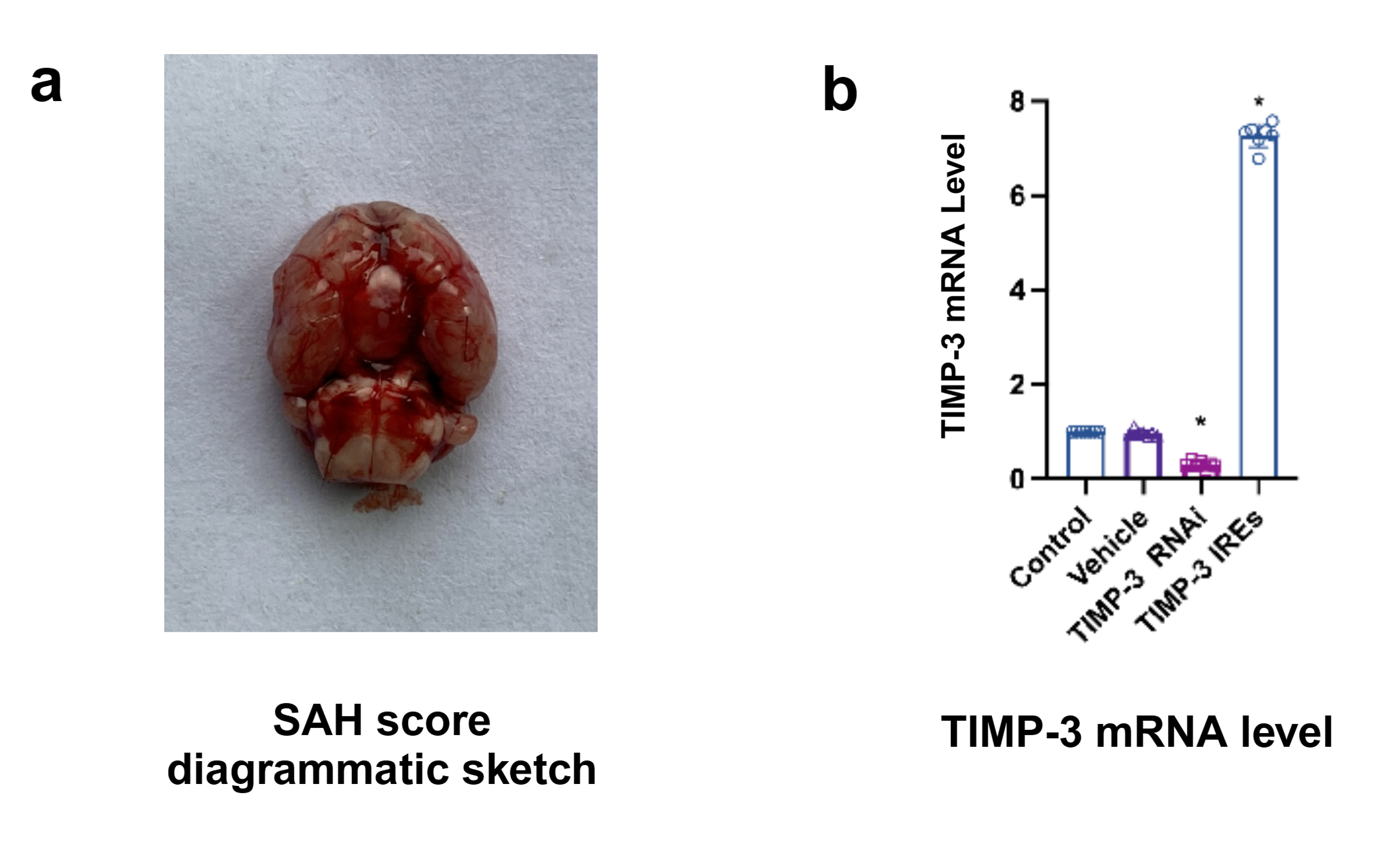

Supplement: Supplementary file 1 — Supplemental Fig. 1 SAH score diagrammatic sketch and TIMP-3 mRNA levels. (a) SAH score diagrammatic sketch. (b) The expression level of TIMP-3 mRNA was detected by PCR (n=6 per group). (* vs. control group; one-way ANOVA, F (3, 24) = 4189, P<0.0001) (TIF 897 KB) [file 10571_2024_1469_MOESM1_ESM.tif]
